# Preparation and Properties of Modified Phenylethynyl Terminated Polyimide with Neodymium Oxide

**DOI:** 10.3390/ma15124148

**Published:** 2022-06-10

**Authors:** Peng Zhang, Hansong Liu, Yilun Yao, Tao Yang, Jinsong Sun, Xiangyu Zhong, Jianwen Bao, Yan Zhao, Xiangbao Chen

**Affiliations:** 1 School of Materials Science and Engineering, Beihang University, Beijing 100191, China; 050131zhangpeng@163.com; 2 AVIC Composite Technology Center, AVIC Composite Corporation Ltd., Beijing 101300, China; liuhansong@foxmail.com (H.L.); m2uyao@sina.com (Y.Y.); ytburning@163.com (T.Y.); sunjsbuaa@163.com (J.S.); xyzhong2003@sohu.com (X.Z.); 3 Science and Technology on Advanced Composites Laboratory, Beijing Institute of Aeronautical Materials, Beijing 100095, China

**Keywords:** phenylethynyl, thermoset polyimide, neodymium oxide, modification, thermo-oxidative stability

## Abstract

Modified phenylethynyl terminated polyimides (PIs) were successfully prepared by using neodymium oxide (Nd_2_O_3_) via high-speed stirring and ultrasonic dispersion methods. In addition, the structure and properties of the Nd_2_O_3_-modified imide oligomers as well as the thermo-oxidative stability of the modified polyimides (PI/Nd_2_O_3_ hybrid) and its modification mechanism were investigated in detail. The thermogravimetric analysis (TGA) results indicated that the 5% decomposition temperature (Td5%) of the PI/Nd_2_O_3_ hybrids improved from 557 °C to 575 °C, which was also verified by the TGA-IR tests. Meanwhile, the weight loss rate of the PI/Nd_2_O_3_ hybrids significantly decreased by 28% to 31% compared to that of pure PI under isothermal aging at 350 °C for 450 h when the added content of Nd_2_O_3_ was between 0.4 wt% and 1 wt%, showing outstanding thermo-oxidative stability. Moreover, the mechanism of the enhanced thermo-oxidative stability for the modified PIs was analyzed by scanning electron microscopy (SEM) and X-ray diffraction (XRD).

## 1. Introduction

Demand for aerospace technology has grown significantly nowadays due to its rapid development, and it now requires materials with better thermo-oxidative stability. Among the various kinds of polymer materials, polyimide (PI) resin and PI matrix composites possess not only outstanding comprehensive properties at high temperatures (280–400 °C), but also the ability to act as load-carrying structures in high temperature environments. In recent years, they have been widely used in aviation, aerospace, and space technology, especially in aero-engines and space aircraft [1,2,3,4].

To improve the thermal-oxidation stability of PIs, one conventional method involves finding changes in the chemical characteristics of PI cross-linked structures. For thermosetting PIs, different types of active terminal groups including nadic anhydride-terminated PIs and phenylethynyl-terminated PIs [5,6,7,8,9,10,11,12,13,14,15,16,17] can provide the specifics of the resin. The thermal decomposition temperature of nadic anhydride-terminated PIs are generally not higher than 510 °C such as PMR-15, PMR-II-50, RP-46, DMBZ-15, etc. [18,19,20]. Compared to the former, phenylethynyl-terminated PIs exhibit better thermo-oxidative stability and process performance, which has always been the research focus in this field. Currently, a new generation of PIs has been developed on this basis [8,9,10,11,12,13] such as PETI-330, PETI-375, AFRPE-4, TriA-X, etc., whose decomposition temperatures are usually between 520 and 550 °C [21,22,23,24]. However, to further improve the thermal-oxidation stability over a long period of time by optimizing the chemical structure [14,15,16,17], a single pathway by chemical modification has been difficult to achieve the requirements of this performance.

To solve this problem, another way of thinking involves physical modification methods, which have also been proposed. Among these methods, various types of inorganic particles have been used (e.g., graphene oxide, aluminum oxide) for the modification of PIs, which play an important role in improving heat resistance [25,26,27,28]. However, the limitation of this method is that many types of particles have a small change in enhancing the thermal performances of the matrix.

Rare earth oxides exhibit outstanding environmental stability [29]. A series of research articles in this field have illustrated that rare earth oxides have a positive effect on the thermal-oxidative stability of polymers such as polyethylene, polyaniline, and thermoplastic PIs. The key sources of the contributing factors of these rare earth oxides involve their distinctive morphologies and catalytic properties [30,31,32,33,34,35,36]. Considering the special atomic structural characteristics of rare earth elements, blending PIs with rare earth oxides is a promising method for developing new organic–inorganic hybrid materials as they may combine the properties of inorganics such as heat resistance with the inherent characteristics of PIs.

Many studies have made advances in the field of rare earth oxides [29,30,31,32,33,34,35,36,37,38,39,40,41,42,43,44]. A typical example involves using them as thermal stabilizers to enhance the thermal stability of polymers [37,38,39]. For the modification of thermoset PIs, most studies have reported on enhancing the tribological properties, thermal conductivity as well as gamma-ray/neutron shielding characteristics using rare earth oxides [40,41]. Nevertheless, when it comes to enhancing the thermal stability of thermoset PIs, there have been a small number of relevant articles that can be searched. The influence of choosing proper rare earth oxides as stabilizers for PIs is significant, as the variety of rare earth oxides is numerous and their properties are quite different. Among all the types of rare earth oxides, light rare earth oxides including praseodymium oxide (Pr_6_O_11_), lanthanum oxide (La_2_O_3_), and neodymium oxide (Nd_2_O_3_) have the potential to be used for polymer modification. Furthermore, studies [42,43,44,45,46] have shown that Nd_2_O_3_ has an obvious effect on the polymer’s thermal stability.

In our previous research, which was inspired by review studies, a low viscosity type of thermoset PIs and composites were modified with Nd_2_O_3_ to improve their thermal oxidation stability [47]. The results showed that the weight loss rates of the modified PI composites dramatically decreased by 25% compared to that of the pure PI composites below 350 °C, after aging for 200 h, when the added content of Nd_2_O_3_ was 1 wt%. However, studies on the influence of the Nd_2_O_3_ weight content on the thermal-oxidation stability are very limited (only 1%, 3%, and 5 wt%), and it could not be ruled out that the most suitable Nd_2_O_3_ content may be below 1 wt%. However, we found that Nd_2_O_3_ could not be uniformly dispersed using the method of high-speed shear dispersion alone, which weakened the thermal oxidation stability. Consequently, in this study, we attempted to use high viscosity PI, combined with high-speed stirring and ultrasonic dispersion methods, to further optimize the amount of Nd_2_O_3_ and obtain better thermal-oxidation stability.

## 2. Materials and Methods

### 2.1. Materials

**Figure 1:**
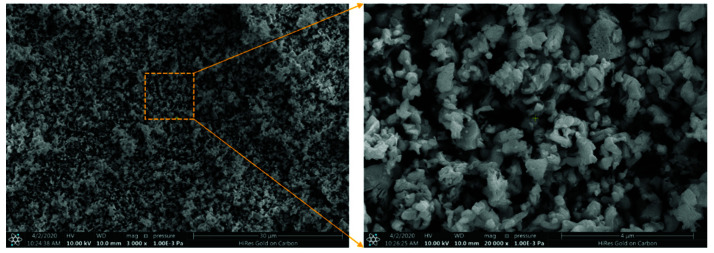
The scanning electron microscope image of Nd_2_O_3_.

In this work, the main monomers used for the synthesis of polyimide resin including 2,2’-bis(trifluoromethyl)benzidine (TFMBZ, 99.95%), 2,3,3,4-biphenyltetracarboxylic dianhydride (α-BPDA, 99.95%), and 4-phenylethynylphthalic anhydride (PEPA, 99.95%) were purchased from Changzhou Sunlight Pharmaceutical Co., Ltd., Changzhou, China, and ethanol (AR) was brought from Beijing Chemical Works, Beijing, China. The scanning electron microscope (SEM) pattern of the neodymium oxide (Nd_2_O_3_, Sinopharm Chemical Reagent Co., Ltd., Beijing, China) particles is shown in Figure 1.

### 2.2. Preparation of Nd_2_O_3_ Modified Polyimide Resin

**Figure 2:**
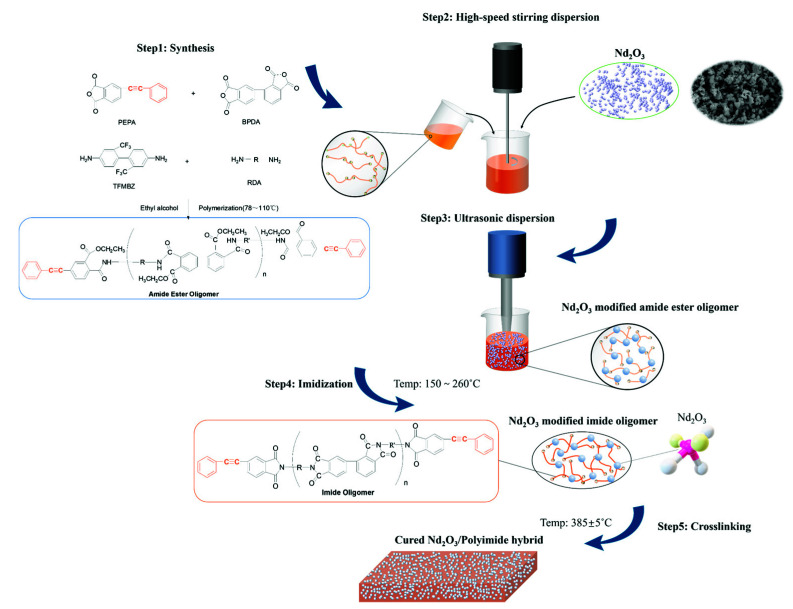
Schematic drawing of the preparation process of the modified polyimide.

The PEPA (236.82 g), α-BPDA (280.69 g), and ethyl alcohol (1207.00 g) were first placed in a 2 L beaker and stirred using a glass bar to form a mixed slurry. The slurry was added to a 3 L three-necked flask that was equipped with a mechanical stirrer, reflux condenser, mercury thermometer, and nitrogen gas inlet. Then, the mixture was heated to reflux (~78 °C) for approximately 1 h to dissolve the anhydrides, followed by an additional 2 h to complete esterification. Then, the TFMBZ (183.32 g) and another diamine (299.18 g) powder were added, and the solution of amide ester oligomers was obtained after stirring and heating from 78 °C to 110 °C for ~2 h. Afterward, the Nd_2_O_3_ powder was added according to the addition ratio (0.2, 0.4, 0.6, 0.8, and 1 wt%), under high-speed stirring at 2000~5000 rpm. Subsequently, the mixture was further dispersed by an ultrasonic disperser to obtain better dispersion, and the Nd_2_O_3_ modified amide ester oligomer was heated up around 150~260 °C to complete imidization. After being converted into an imide oligomer, it was then cured at 385 ± 5 °C for 5 h and formed a modified Nd_2_O_3_/PI hybrid. The synthesis process of the Nd_2_O_3_/PI hybrid is illustrated in Figure 2.

### 2.3. Characterization

The Fourier transform infrared (FTIR) spectra of the modified imide oligomers and Nd_2_O_3_/PI hybrids with different content Nd_2_O_3_ were recorded using a Nicolet S50 FTIR spectrophotometer. The X-ray diffraction (XRD) patterns of the Nd_2_O_3_ modified imide oligomer with varied Nd_2_O_3_ content as well as unmodified PI and Nd_2_O_3_/PI hybrids were recorded using a Bruker D8 advance diffractometer (Germany). The diffractogram was measured at an operating 2q range of 2θ = 3–60°. A SEM was used to investigate the sample shape characteristics using a field emission SEM (FE-SEM, Hitachi S-4800), and a rheology test was used to study the rheological properties of the pure imide oligomer and Nd_2_O_3_-modified imide oligomers using a TA Instruments AR 2000 rheometer. The oligomer powders were pressed into thin wafers, 2.5 cm in diameter, and tested at a heating rate of 2 °C/min from 260 °C to 400 °C. The glass transition temperature (T_g_) of PIs was measured through dynamic mechanical thermal analysis (DMA) using a TA Instruments DMAQ800 dynamic mechanical analyzer from room temperature (RT) to 500 °C at a heating rate of 5 °C/min, under a frequency of 1 Hz, and using the double cantilever deformation mode. Thermogravimetric analysis (TGA) was performed with a NETZSCH STA 449F5 at a heating rate of 10 °C/min from 40 to 800 °C under air purging. The TGA-FTIR studies were carried out using a NETZSCH STA 449F5 thermogravimetric analyzer that was interfaced with a TENSORII FT-IR spectrometer. Thermal oxygen aging was processed in a high temperature forced air oven, and the Nd_2_O_3_/PI hybrids and pure PI samples were cut into a size of 80 mm × 30 mm × 4 mm. First, the samples were heated to 200 °C and held for 1 h to remove the moisture. Then, the sample weights (recorded as M_0_) were recorded by an analytical balance, with a precision of 0.001 g. After that, the samples were placed back into the oven and heated to 350 °C and aged for 450 h. The samples were removed at different times for weighing and recorded as M_t_. Accordingly, the weight loss rate at time t was calculated as follows:

Δ M_t_ = (M_0_−M_t_)/M_0_ × 100%.

## 3. Results and Discussion

### 3.1. Structure Characterization of Nd_2_O_3_ Modified Imide Oligomer

**Figure 3:**
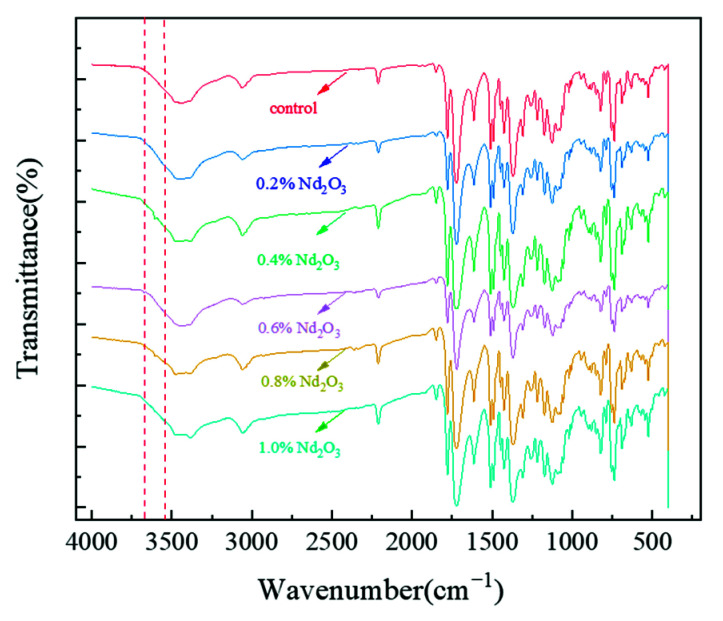
The FTIR spectra of the imide oligomers with different Nd_2_O_3_ content.

The FTIR spectroscopy experiments were performed to investigate whether Nd_2_O_3_ was successfully added into the imide oligomer and the interactions between them (Figure 3). The characteristic absorption bands related to the pure imide oligomer showed obvious absorption peaks arising from the asymmetric and symmetric stretching vibrations of the C=O groups in the imide rings at around 1779 and 1722 cm^−1^. Another characteristic absorption band of the imide group derived from C–N stretching appeared at approximately 1369 and 738 cm^−1^. In addition, the band at 2212 cm^−1^ was related to the stretching vibrations of the phenylethynyl group (C≡C). Thus, the results indicated that the imide oligomer formed through thermal imidization. The 0.2, 0.4, 0.6, 0.8, and 1 wt% Nd_2_O_3_ content modified oligomers (Nd_2_O_3_/oligomer) showed a relatively weak absorption peak at 3605 cm^−1^, which corresponded well to the O–H of Nd(OH)_3_, and we preliminarily inferred that the Nd_2_O_3_ present in the oligomers was in the form of Nd(OH)_3_ [46,47,48].

**Figure 4:**
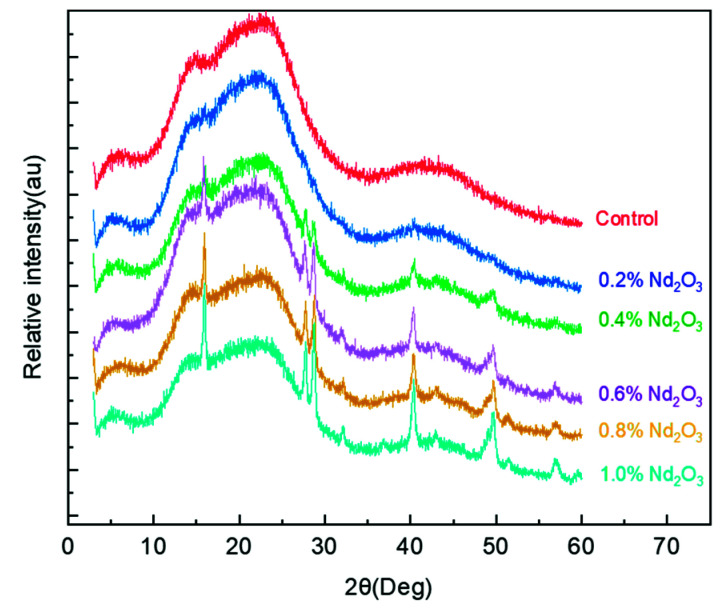
The XRD spectra of the imide oligomers with different Nd_2_O_3_ contents.

The form and dispersion states of Nd_2_O_3_ in the imide oligomers were further assessed by XRD characterization. Figure 4 shows the XRD patterns of the imide oligomer and Nd_2_O_3_-modified imide oligomer. In the XRD pattern, a broad amorphous diffraction peak appeared between 10° and 30° in all of the samples, indicating that the imide oligomer substrate was amorphous. This was probably because the molecular chains of the oligomers were irregular, as they were composed of low-weight organic atoms, leading to the formation of amorphous morphological structures. The XRD patterns of the Nd_2_O_3_ modified oligomer hybrids showed several sharp diffraction peaks centered at approximately 15.9°, 27.7°, 28.7°, 32.1°, 40.3°, 42.9°, 49.6°, 51.3°, and 56.9° when the content of Nd_2_O_3_ was greater than 0.2 wt%. Moreover, with the increase in the Nd_2_O_3_ content, the intensity of the relevant crystallization absorption peaks of modified oligomers increased significantly, which can probably be attributed to the crystallization characteristics of neodymium oxide. These peaks were found to be related to Nd(OH)_3_ (ICDD PDF 06-0601) after indexing in the ICDD PDF2 database. The formation of Nd(OH)_3_ was due to the hydration of Nd_2_O_3_ during the imidization reaction [46,47,48], indicating that Nd_2_O_3_ was successfully added into the imide oligomer in a specific chemical form. Consequently, the formed Nd(OH)_3_ might react with the PI matrix or a byproduct during the subsequent curing process to form a new structure. Additionally, the preparation of modified oligomers with different Nd_2_O_3_ contents (0.2, 0.4, 0.6, 0.8, and 1 wt%) was used to identify the optimal content of Nd_2_O_3_, for which the modified PI would obtain the best thermal oxidation stability.

**Figure 5:**
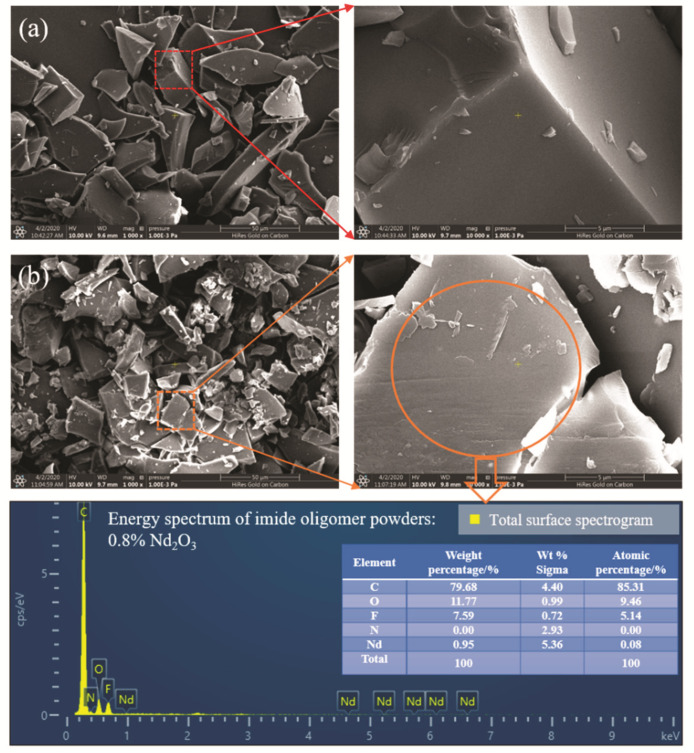
The SEM pattern of the imide oligomer powders: (**a**) control and (**b**) 0.8% Nd_2_O_3_.

The FTIR and XRD results already demonstrated that Nd_2_O_3_ changed to the chemical structure of neodymium hydroxide after addition to the imide oligomers. To further demonstrate the physical morphology of neodymium hydroxide in the oligomers, the micro morphologies of the modified imide oligomer powders were observed by SEM, as shown in Figure 5. A comparison of the surface morphology between the pure imide oligomer and 0.8% Nd_2_O_3_-modified oligomers revealed no significant differences. The presence of neodymium hydroxide particles could not be directly observed on the surface of the modified oligomers; however, the existence of neodymium elements could be found after energy spectrum analysis. Consequently, we inferred that Nd(OH)_3_ was dissolved in the imide oligomers after the imidization reaction.

**Figure 6:**
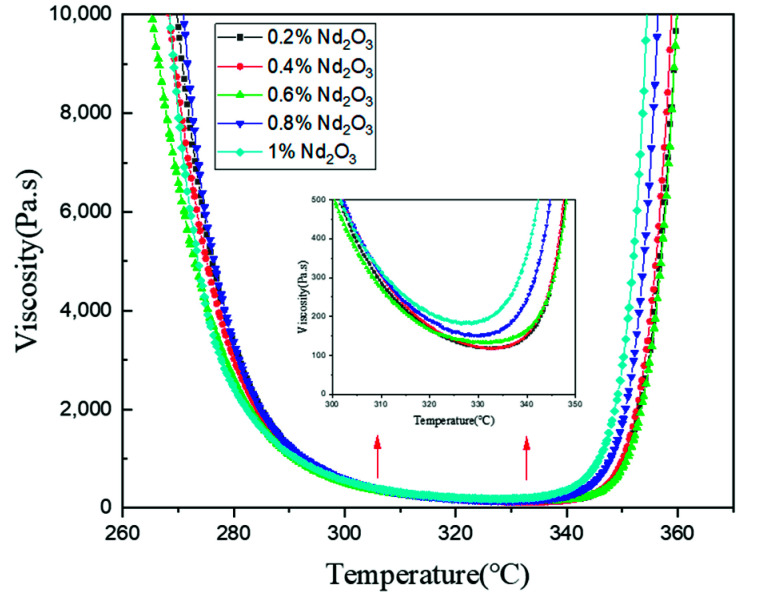
The rheological properties of oligomers with different Nd_2_O_3_ contents.

A rheology test was used to study the effect of Nd_2_O_3_ content on the rheological properties. As shown in Figure 6, the minimum viscosities of the modified oligomers with 0.2, 0.4, 0.6, 0.8, and 1 wt% Nd_2_O_3_ were 118.6, 119.0, 132.5, 150.7, and 182.6 Pa·s, respectively. We found that the lowest viscosity of the modified oligomers increased gradually and the temperature of the lowest viscosity decreased slowly with increasing Nd_2_O_3_ content, which contributed to the adsorption of imide oligomers by the nano Nd_2_O_3_ particles and reduced the motility of the oligomeric molecular chains. The higher the content of neodymium oxide, the more obvious the impact on the rheological properties of the modified oligomers. However, because the mass fraction of Nd_2_O_3_ was less than 1 wt%, the effect of neodymium oxide on the viscosity would not significantly change the process performance.

### 3.2. Thermomechanical Properties of Modified Polyimides

**Figure 7:**
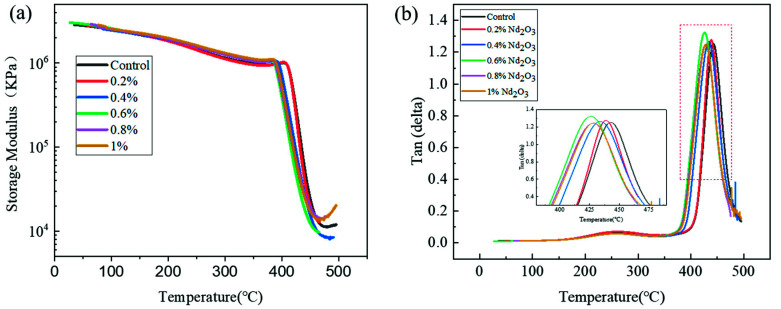
The DMA curve of the polyimide with different contents of Nd2O3: (**a**) storage modulus and (**b**) tan δ.

The DMA results of the PIs are shown in Figure 7. According to the diagram, after the addition of Nd_2_O_3_, the glass transition temperature (Tg) of the PI polymer declined. Moreover, for the modified PI, with increasing Nd_2_O_3_ content, the T_g_ value showed an overall downward trend. These results showed evidence that Nd_2_O_3_ hindered collisions among the reactive crosslinking groups, thus blocking the movement of the oligomers and resulting in the decreased density of cross-linked modified PI(PI/Nd_2_O_3_ hybrid) and reduced T_g_. With increasing Nd_2_O_3_ content, more hindered collisions occurred and the T_g_ of the PI/Nd_2_O_3_ hybrid declined gradually. However, as the addition of Nd_2_O_3_ was not more than 1 wt%, the neodymium oxide would not significantly worsen the Tg of the modified polyimide.

### 3.3. Thermal Decomposition Properties of the Modified Polyimides

**Figure 8:**
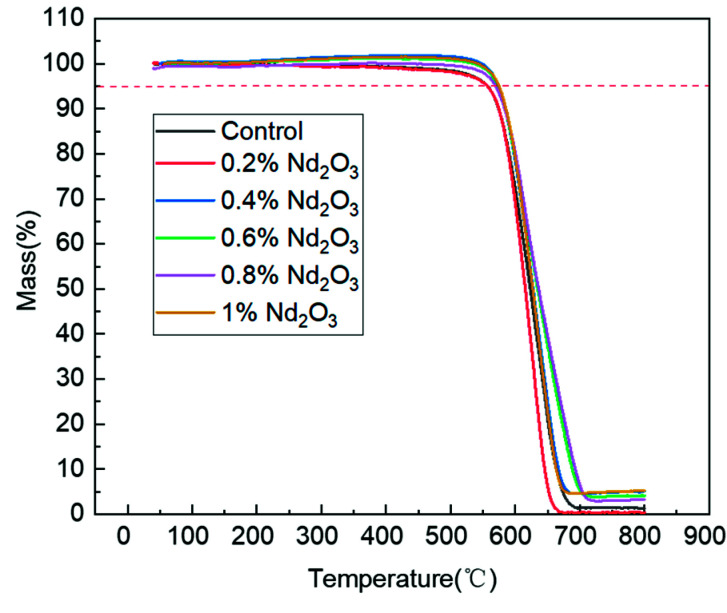
The TGA curves of the polyimides with different Nd_2_O_3_ contents.

To study the effect of Nd_2_O_3_ enhancement on the thermal stability of the PI/Nd_2_O_3_ hybrids, TGA was performed in air in the temperature range of 40–800 °C. The TGA curves of the pure PI and PI/Nd_2_O_3_ hybrids are illustrated in Figure 8. For the thermal stability of the pure PI and PI/Nd_2_O_3_ hybrids, the 5% weight-loss temperature (T_d5%_) and 10% weight-loss temperature (T_d10%_) are listed in Table 1. The results revealed that the PI/Nd_2_O_3_ hybrids exhibited a higher decomposition temperature than that of pure PI, and the T_d5%_ values of these hybrids appeared to improve with an increase in the Nd_2_O_3_ content. With increased Nd_2_O_3_ loading from 0 to 0.4 wt%, the T_d5%_ of the PI/Nd_2_O_3_ hybrids increased from 557 °C to 574 °C, while the T_d10%_ of the PI/Nd_2_O_3_ hybrids increased from 574 °C to 584 °C. When the Nd_2_O_3_ loading exceeded 0.4 wt%, the T_d5%_ and T_d10%_ improved slowly. This meant that Nd_2_O_3_ could prevent the thermal oxygen degradation of the PI matrix with low filler content. Meanwhile, the enhanced thermal stability of the PI/Nd_2_O_3_ hybrid would possibly depend on the Nd_2_O_3_ bonds physically or chemically interacting with the PI matrix. The mechanism was further assessed by XRD.

**Figure 9:**
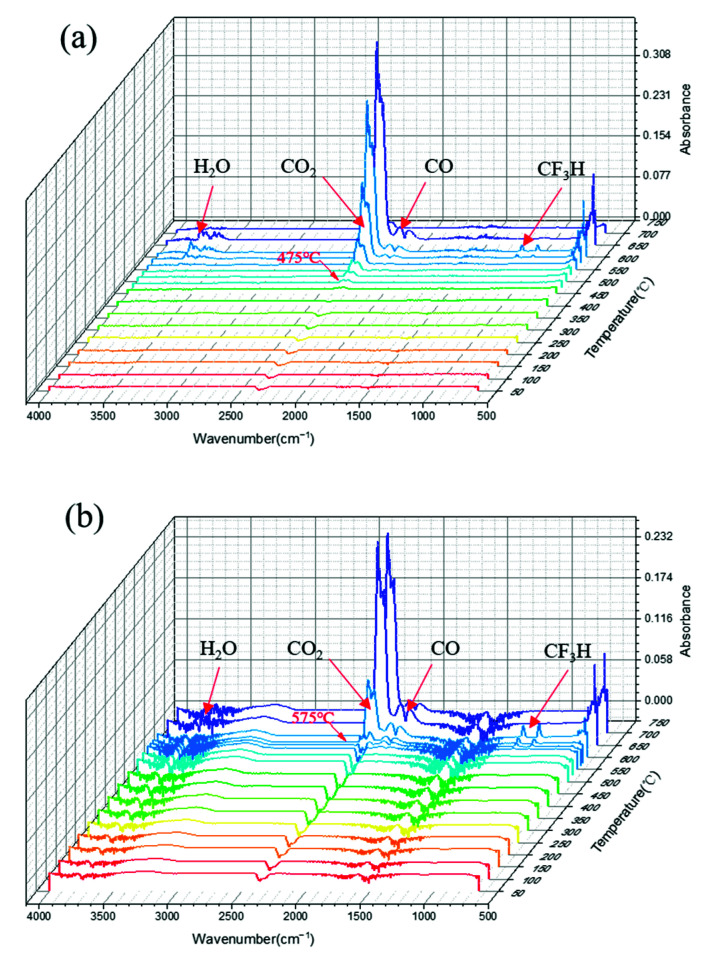
The release of degradation products was examined by TGA-FTIR at different temperatures: (**a**) control and (**b**) 0.4% Nd_2_O_3_.

The weight loss during the heating process was definitely due to the escape of gaseous products, which resulted from the fracture of PI molecule chains. To further investigate the thermal degradation mechanisms of the pure PI and PI/Nd_2_O_3_ hybrids, the release of the degradation products was examined by TGA-FTIR under an air atmosphere at different temperatures. A stacked plot of the FTIR spectra of the byproducts that escaped from TGA during the degradation process is illustrated in Figure 9, and the following characteristic bands were observed during the pyrolysis of PI. The bands in the range of 3500–3800 cm^−1^ were associated with the stretching of O–H and were derived from H_2_O; the peaks at around 2360 and 2322 cm^−1^ were characteristic bands of CO_2_; the characteristic double bands (2180 and 2110 cm^−1^) belonged to CO; and the bands at approximately 1152 cm^−1^ were assigned to the symmetric stretching vibrations of F–C–F, arising from CF_3_H, which were only significantly observed when the temperature was above 550 °C and completely disappeared at 650 °C. These results indicate that the major thermal degraded products were CO_2_, CO, H_2_O, and CF_3_H. Meanwhile, the CO_2_ was the predominant gaseous byproduct throughout the thermal degradation process under the oxygen atmosphere. Additionally, we found that the pure PI and PI/Nd_2_O_3_ hybrids had similar volatilized products, except for the temperature at which volatilized products begin to be generated. It was certainly clear that the CO_2_ of the PI/Nd_2_O_3_ hybrids was observed at 575 °C (Figure 9b), which was much higher than that of pure PI (Figure 9a). The intensity of volatilized CO_2_ peaks increased slowly from 475 °C to 550 °C, whereas it was found to increase rapidly when the temperature exceeded 550 °C. We concluded that the PI/Nd_2_O_3_ hybrids exhibited a significantly better thermal decomposition temperature compared to the pure PI, which corresponded to the TGA results.

### 3.4. Thermal Oxidative Stability of the Modified Polyimides

**Figure 10:**
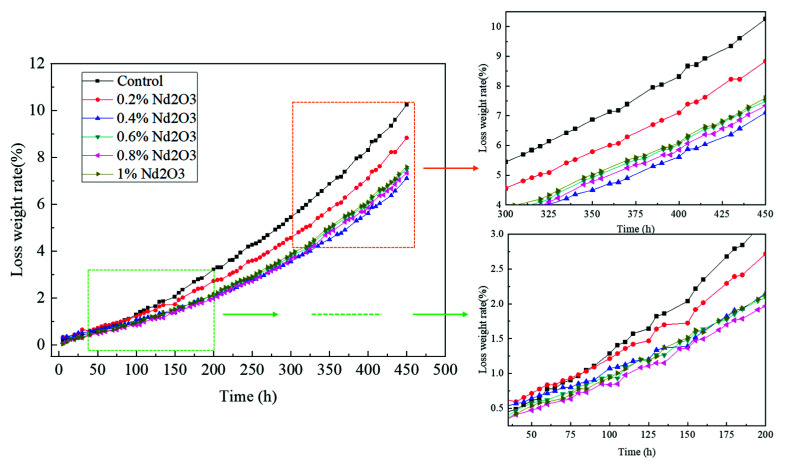
The weight loss rate of the polyimide with different contents of Nd_2_O_3_ at 350 °C.

To better study the thermal oxidative stability of the Nd_2_O_3_-modified PIs in a long-term service environment, the weight loss changes of the pure PI and PI/Nd_2_O_3_ hybrids were evaluated by an isothermal thermo-oxygen aging method. The weight loss rates of the modified PIs with different Nd_2_O_3_ contents during isothermal aging at 350 °C for 450 h are shown in Figure 10. The weight loss rates of both the PI/Nd_2_O_3_ hybrids and pure PI increased with prolonged aging time. However, the weight loss rates of the PI/Nd_2_O_3_ hybrids were notably lower than that of the pure PI. When the aging time was 450 h, the weight loss rates of the PI/Nd_2_O_3_ hybrids at 0, 0.2, 0.4, 0.6, 0.8, and 1 wt% were 10.25, 8.84, 7.11, 7.23, 7.04, and 7.29%, respectively. Thus, the weight loss rates of the 0.2, 0.4, 0.6, 0.8, and 1 wt% samples decreased by 13.8, 30.6, 29.5, 31.3, and 28.9%, respectively, compared to the pure PI. We concluded that the thermal-oxidative stability of the modified PIs was dramatically better than that of the pure PIs when the Nd_2_O_3_ content was between 0.4 wt% and 1 wt%, which was similar to the TGA results.

**Figure 11:**
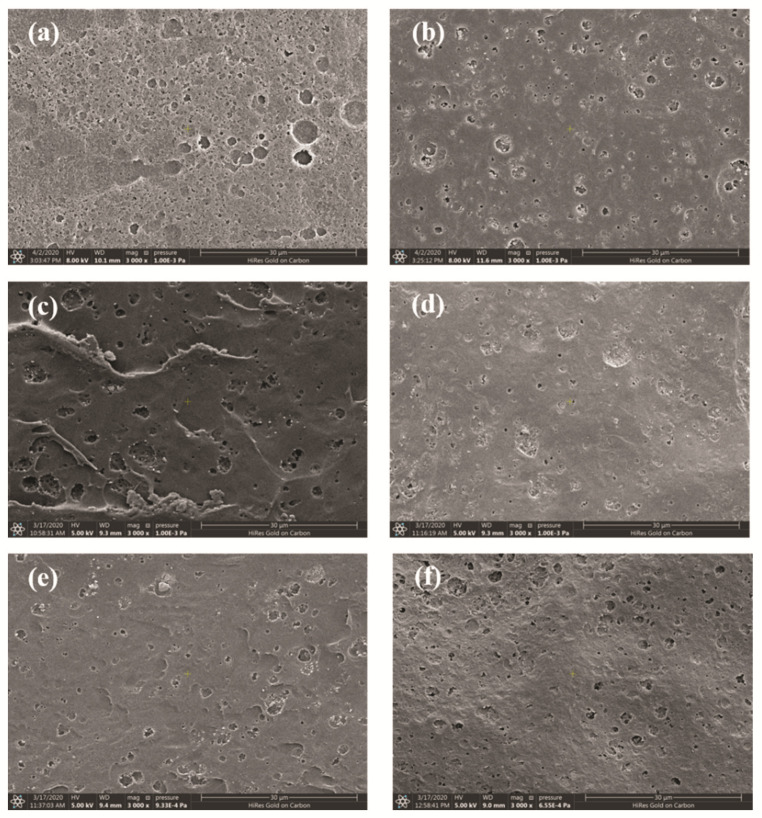
The SEM patterns of the resin surface after isothermal aging at 350 °C for 450 h: (**a**) control; (**b**) 0.2 wt% Nd_2_O_3_, (**c**) 0.4 wt% Nd_2_O_3_, (**d**) 0.6 wt% Nd_2_O_3_, (**e**) 0.8 wt%, and (**f**) 1 wt% Nd_2_O_3_.

**Figure 12:**
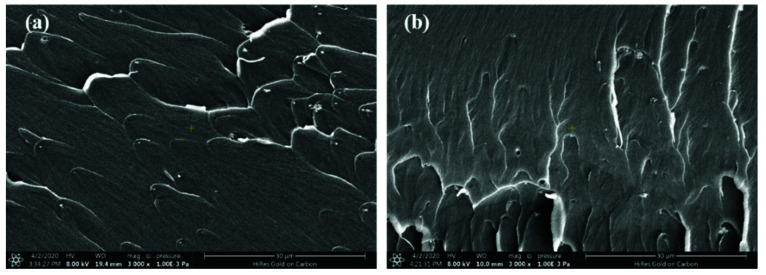
The SEM patterns of the resin section after isothermal aging at 350 °C for 450 h: (**a**) control and (**b**) 0.8 wt% Nd_2_O_3_.

As shown in Figure 11, the surface morphologies of the modified PI resin with different Nd_2_O_3_ contents (0, 0.2%, 0.4%, 0.6%, 0.8%, and 1%) after isothermal aging at 350 °C for 450 h were characterized by SEM. We found that after prolonged high temperature aging, numerous pores were generated on the surface of the unmodified PI resin. The structure and size of the pores were randomly distributed, and the density of the pores was high. The pores presented a semi-continuous state. However, the surface porosity of the Nd_2_O_3-_modified PI resin was significantly reduced, and the pore size was slightly larger than that of the unmodified PI, while the density of the pores was significantly reduced. Conversely, the internal structure (Figure 12) of the PI and modified PIs was at a distance away from the aerobic area of the surface, while the intact microstructure and accumulation density were still maintained, and the pores caused by resin decomposition were not observed. Additionally, the enhancement of the thermal-oxidation stability was due to the formation of Nd_2_O_3_/PI hybrids, which could inhibit the diffusion of oxygen and reduce the degradation of the PI resin.

**Figure 13:**
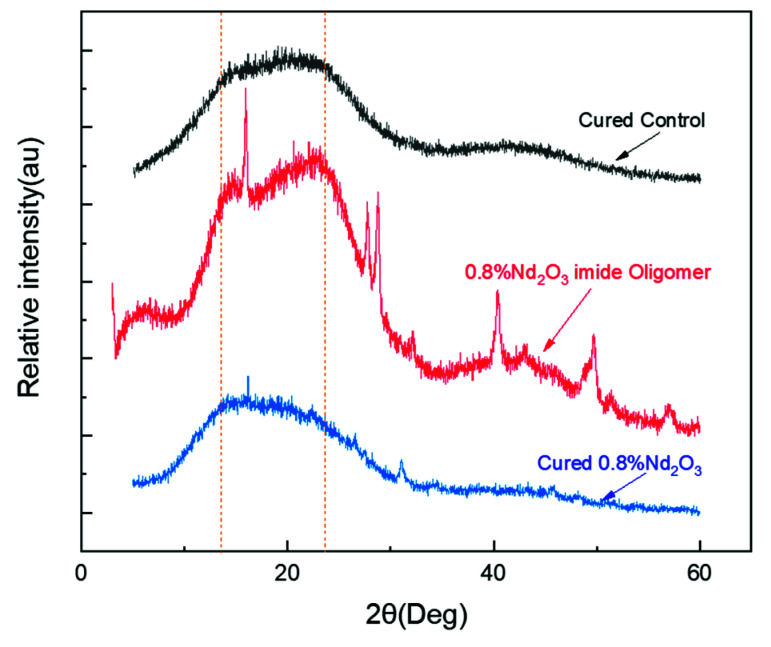
The XRD spectra of the Nd_2_O_3_/PI hybrids (0.8 wt%) compared to the modified imide oligomer.

For the enhancement mechanism of thermal-oxidation stability, XRD analysis was carried out on the pure PI and Nd_2_O_3_-modified imide oligomer (0.8 wt%), while the Nd_2_O_3_/PI hybrids (0.8 wt%) were used to investigate the interactions between Nd_2_O_3_ and PI, as shown in Figure 13. A broad amorphous diffraction peak between 10° and 30° was found from the XRD patterns of the cured, pure PI, showing its amorphous structure. The characteristic diffraction peak of Nd(OH)_3_ was found in the modified imide oligomer (0.8 wt%), which meant that the Nd_2_O_3_ was converted into Nd(OH)_3_. The XRD patterns of the Nd_2_O_3_/PI hybrids (0.8 wt%) were almost the same as that of pure PI. The disappearance of the characteristic diffraction peak for Nd(OH)_3_ obviously demonstrated that Nd(OH)_3_ was chemically incorporated into the PI resin matrix and formed the Nd_2_O_3_/PI hybrids. This was certainly attributed to the reactivity of the hydroxyl group in Nd(OH)_3_, which led to an increase in the interactions between the Nd_2_O_3_ and PI matrix during the curing process. The formation of Nd_2_O_3_/PI hybrids could inhibit the diffusion of oxygen molecules in the crosslinked PI resin structure, which was attributed to strong interaction between the neodymium oxide and the polyimide matrix during the curing process. Consequently, this slowed down the reaction activity of the oxygen molecules, thus hindering the fracture of the crosslinked chains caused by thermal oxygen decomposition, resulting in the enhancement of the thermal-oxidation stability of PI [47,48].

## 4. Conclusions

The phenylethynyl terminated PIs modified with various Nd_2_O_3_ contents were successfully prepared by high-speed stirring and ultrasonic dispersion methods, and the thermal-oxidation stability of the pure PI and modified PIs (PI/Nd_2_O_3_ hybrids) was evaluated by TGA-IR and the isothermal thermo-oxygen aging methods. The TGA results indicate that the T_d5%_ of the PI/Nd_2_O_3_ hybrids improved from 557 °C to 575 °C when the added amount of Nd_2_O_3_ in the modified PIs was between 0.4 wt% and 1 wt%. Meanwhile, the evolved gas was analyzed by TGA-IR, which showed that CO_2_ was the dominant degradation product. Furthermore, the weight loss rate of the PI/Nd_2_O_3_ hybrids was significantly reduced by 28% to 31% after isothermal thermo-oxygen aging at 350 °C for 450 h compared to that of pure PI, when the content of Nd_2_O_3_ was between 0.4 wt% and 1 wt%. This corresponded to the TGA results and indicated a dramatically higher thermal oxidation stability. The outstanding thermo-oxidative stability was attributed to the chemical incorporation of Nd_2_O_3_ into the PI matrix, which could inhibit oxygen diffusion and slow down PI resin degradation due to the characteristics of the Nd elements.

## Figures and Tables

**Table 1:** The TGA database of the polyimide with different Nd_2_O_3_ contents (Td5%, Td10%).

Content of Nd_2_O_3_/%	T_d5%_ (Air)/°C	T_d10%_ (Air)/°C
0	557	574
0.2	557	574
0.4	574	584
0.6	573	584
0.8	570	584
1.0	575	585

## Data Availability

The data presented in this study are available upon request from the corresponding author. The data are not publicly available due to the fact that they are also part of an ongoing study.
